# Perception and Social Impact of Blackboard-Based Online Learning in a Psychology Course: Al-Qunfudhah Medical Students’ Opinion

**DOI:** 10.7759/cureus.68822

**Published:** 2024-09-06

**Authors:** Anas Sarhan

**Affiliations:** 1 Department of Internal Medicine, College of Medicine, Umm Al-Qura University, Makkah, SAU

**Keywords:** al-qunfudah, online learning, perception, psychology, social health

## Abstract

Background: Online learning was considered a novel method of teaching that affects university lifestyle and health. This study focused on online perception of Al-Qunfudhah medical students during psychology course learning and the effect of online teaching on social life.

Methods: Fifth-year students participated in an online psychology course. The online questionnaire consisted of 15 questions: three for demographic distribution, seven to assess online perception, and five to measure the effect of online teaching on students’ social health. Questionnaire validity was checked by a preliminary pilot study, and Cronbach's alpha was used to assess internal consistency. A p-value less than 0.05 was considered significant.

Results: Seventy-five (83.3%) students participated in the study: 48 (64%) female and 27 (36%) male. Fifty-eight (77.3%) medical students documented a good perception of online learning of a psychology undergraduate course, whereas 17 (22.7%) had a poor perception. Additionally, 29 (78.4%) female students reported online teaching had a significant effect on their social and psychological health (p < 0.010), whereas eight (21.6%) male students showed that online teaching does not affect their social life.

Conclusion: Al-Qunfudhah medical students, both men and women, highly participated in the psychology course online learning; however, female students were socially and psychologically affected by online learning more than male students.

## Introduction

Online teaching can be defined as interactive education through the internet with the use of electronic devices [1]. Students all over the world use some form of online technologies, including Blackboard, Cloud Meetings, Zoom, and Microsoft Teams, as tools for online learning. Many universities and colleges offer online teaching in the educational process because it covers a large population with a less expensive option [2,3]. Because of the COVID-19 pandemic, many universities were pushed to online learning to provide students with an educational environment other than face-to-face learning [4]. Many colleges are faced with two challenges when shifting from classroom attendance to online teaching: which online platform to use and what is the availability of internet access with suitable speed to display online teaching [5]. Online and face-to-face learning reveal some similarities. In both, students have to learn new subjects, attend classes, share in groups, and submit assignments. However, one study documented several differences in the results between traditional learning and online learning [6].

According to University College of London program of medical education that was applied in Al-Qunfudhah College of Medicine, Umm Al-Qura University, Saudi Arabia, the psychology course for undergraduates was delivered in a combination of interactive lectures, seminars, clinical classes, and problem-based learning (PBL), with two lectures of two hours per week. In spite of the availability of Blackboard online teaching as an effective tool in online teaching, a recent model of online learning exposed the students during the COVID outbreaks to some sort of psychological stress, even well-prepared students need psychological support [7]. Furthermore, a recent model of online learning exposed the students during the COVID-19 outbreaks to some sort of psychological stress, even well-prepared students need psychological support [7]. The aim of this study was to measure the perception of Al-Qunfudhah medical students to online teaching of an undergraduate psychology course and the effect of online teaching on their social life.

## Materials and methods

This nonrandomized cross-sectional study was performed on the medical students at Al-Qunfudhah Medical College, Umm Al-Qura University, Saudi Arabia. All the fifth-year students studying psychology were involved. The students in this course study all aspects of medical psychology in 12 weeks, including Introduction to Psychiatry; Psycho-Pathology; Psychotic Disorders I, II, & III; Mood Disorders I, II, & III; Anxiety Disorders I, II, & III; Biopsychosocial Model; Psychiatric History; Mental State Examination; Classification System in Psychiatry; Clinical Sessions in Psychotic Disorders; Clinical Sessions in Bipolar Disorders; video sessions in anxiety disorders; and video sessions in depressive disorders. Umm AL-Qura University, like most of the universities of Saudi Arabia, adopted online teaching on Blackboard (established by the University of Botswana in 2002). An online questionnaire was constructed in English and delivered to the students through social media (WhatsApp) from April to June of 2024, after the end of the course. Most of the students from the fifth year, both men and women, were included in our study (n = 75), all of whom took the study seriously. Exclusion criteria included the students who failed to fill in the consent form and first-, second-, third-, fourth-, and sixth-year students. Our questionnaire comprised four sections. The first section included demographic distribution (gender, education level, and Grade Point Average (GPA)); the second section included seven questions measuring the perception of students toward online teaching; the third section contained five questions about the psychological impact of Blackboard online learning on the students. The questionnaire was constructed on Google Drive (Google, Mountain View, CA), and each question answer was based on a Likert scale with five answers (1 = strongly disagree, 2 = disagree, 3 = neutral, 4 = agree, and 5 = strongly agree). The sample size was calculated using the Raosoft program. With a confidence interval level of 95% and 5% margin of error, the minimum sample size required is 76. We received 75 completed questionnaires.

Statistical analysis

The data from Google Drive were tabled in an Excel sheet (Microsoft Corporation, Redmond, WA) to be analyzed by Statistical Package for the Social Sciences (version 27.0, IBM Corp., Armonk, NY). The categorical data (including demographics, student perception of Blackboard, and psychological effect of Blackboard on students) were expressed as frequencies and percentages. Moreover, a Chi-squared test was used to correlate between the sociodemographic data and the perception of students and the psychological effect. The results of the Chi-squared test were presented in frequencies, percentages, and p-value, with p-value < 0.05 considered statistically significant.

## Results

Sociodemographic distribution

Of the 90 students approached to participate in our study, 83.3% (n = 75) of them shared in the questionnaire; there were 48 (64%) female students and 27 (36%) male students. Regarding students’ GPA, a majority of them had a GPA between 2.5 and 3 (24, 32%) and 3 and 3.5 (24, 32%; Table 1).

**Table 1:** Sociodemographic data (n = 75)

Parameter	Category	N	%
Gender	Male	27	36.0
	Female	48	64.0
Educational level	Fifth year	75	100.0
GPA	Less than 2	2	2.7
	Between 2 and 2.5	7	9.3
	Between 2.5 and 3	24	32.0
	Between 3 and 3.5	24	32.0
	Between 3.5 and 4	18	24.0

Students’ perception of Blackboard-based online teaching

**Figure 1:**
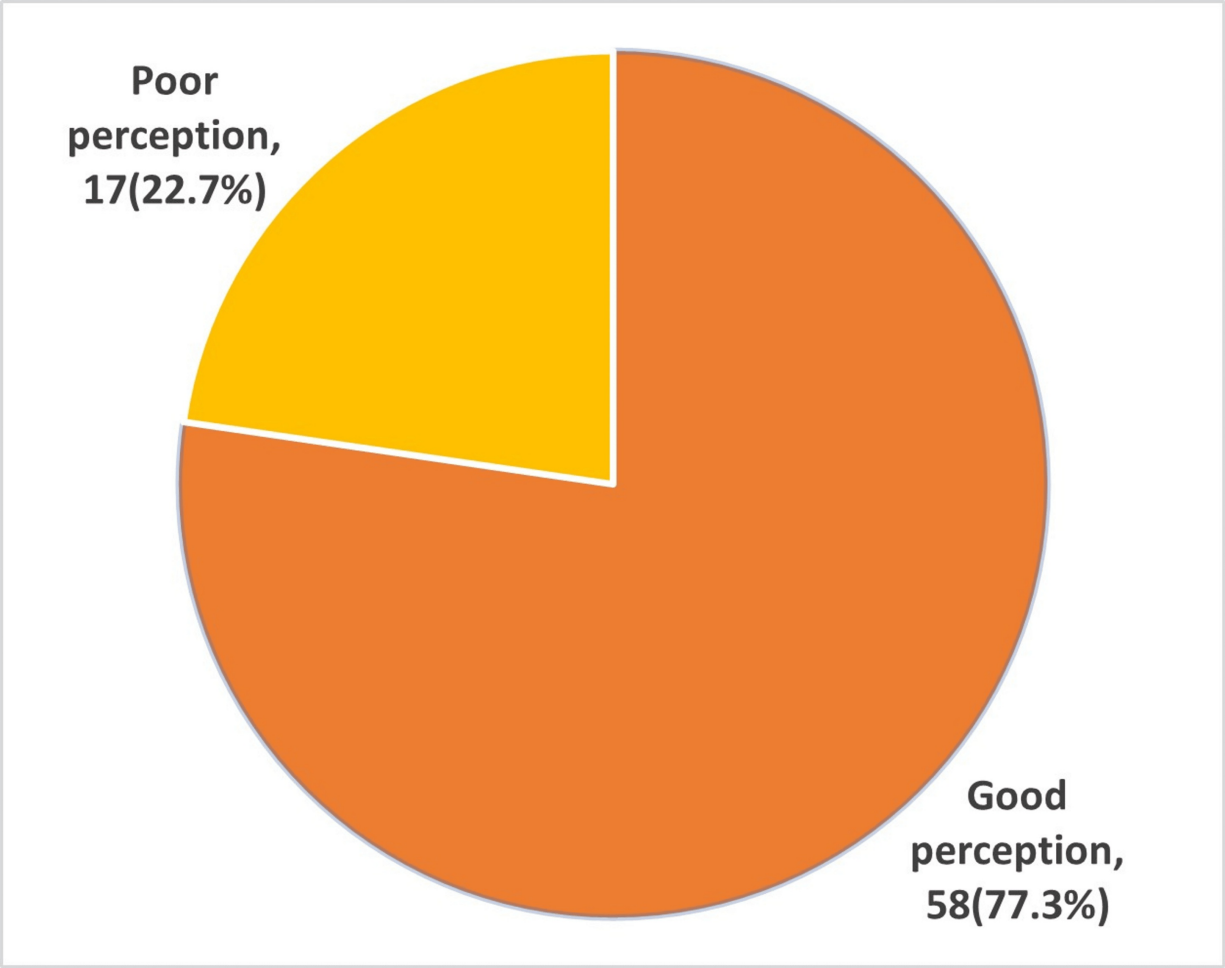
Overall perception of medical students toward online psychology courses

Our study investigated medical students’ perception of Blackboard teaching during the study of a psychology course. Most of the students, 54 (58.7%), agreed that online teaching is motivating. A large number of them, 42 (56%), decided that it is very easy to be engaged in lessons. Forty-three (57.3%) students were able to ask questions during the lecture. A large number, 39 (52%), of the students enjoyed online learning, and 41 (54.6%) found it to be interactive. Furthermore, the results of the questionnaire gave a near-equal response when the students were asked about the effectiveness of online teaching versus face-to-face teaching: 25 (33.3%) of them disagreed and 27 (36%) agreed. Thirty-five (46.7%) students documented that the staff member was well-prepared for the session (Table 2, Figure 1). Results of the medical students’ overall perception showed that 58 (77.3%) had a good perception of psychology eLearning and 17 (22.7%) had a poor perception (Figure 1). Good perception of students to online teaching open the door to be merged it with in-person education as a hybrid method of teaching.

**Table 2:** Respondents’ perceptions toward online learning and teaching experiences (n = 75) N (%), Pearson’s Chi-squared test.

Parameter	Category	N	%	p-value
Q1. Online learning is very motivating.	Strongly disagree	7	9.3	0.31
	Disagree	8	10.7	
	Neutral	16	21.3	
	Agree	30	40.0	
	Strongly agree	14	18.7	
Q2. It is very easy to engage in psychology online lectures.	Strongly disagree	8	10.7	0.92
	Disagree	9	12.0	
	Neutral	16	21.3	
	Agree	22	29.3	
	Strongly agree	20	26.7	
Q3. I ask questions very easily.	Strongly disagree	7	9.3	0.27
	Disagree	9	12.0	
	Neutral	16	21.3	
	Agree	27	36.0	
	Strongly agree	16	21.3	
Q4. Psychology eLearning is very enjoyable.	Strongly disagree	9	12.0	0.31
	Disagree	10	13.3	
	Neutral	17	22.7	
	Agree	21	28.0	
	Strongly agree	18	24.0	
Q5. I would like psychology eLearning to be interactive.	Strongly disagree	5	6.7	0.21
	Disagree	5	6.7	
	Neutral	24	32.0	
	Agree	25	33.3	
	Strongly agree	16	21.3	
Q6. I feel that psychology online teaching is effective as traditional teaching.	Strongly disagree	13	17.3	0.12
	Disagree	12	16.0	
	Neutral	23	30.7	
	Agree	16	21.3	
	Strongly agree	11	14.7	
Q7. The medical staff was well prepared for online learning.	Strongly disagree	7	9.3	0.36
	Disagree	7	9.3	
	Neutral	26	34.7	
	Agree	17	22.7	
	Strongly agree	18	24.0	

Psychological impact of online teaching on medical students

Most of the students, 47 (62.7%), suffer from headache and vision problems because of online teaching. Forty-eight (64%) students suffer from anxiety, and 43 (57.3%) complain about stress during online teaching. Meanwhile, 43 (57.3%) students feel socially isolated during online learning. Regarding physical activity, a large number of them, 41 (54.7%), were unable to do physical activities during Blackboard teaching (Table 3).

**Table 3:** Impacts of online learning and teaching on students’ health and social life (n = 75) N (%), Pearson’s Chi-squared test, * p < 0.05 (significant).

Parameter	Category	N	%	p-value
Q8. Headache and blurry vision were considered one of the complications of psychology online teaching.	Strongly disagree	6	8.0	0.23
	Disagree	3	4.0	
	Neutral	19	25.3	
	Agree	24	32.0	
	Strongly agree	23	30.7	
Q9. Anxiety during psychology eLearning was considered one of its complications.	Strongly disagree	6	8.0	0.13
	Disagree	5	6.7	
	Neutral	16	21.3	
	Agree	27	36.0	
	Strongly agree	21	28.0	
Q10. Stress was one of the social drawbacks of psychology online learning.	Strongly disagree	9	12.0	0.07
	Disagree	6	8.0	
	Neutral	17	22.7	
	Agree	21	28.0	
	Strongly agree	22	29.3	
Q11. Social isolation was considered one of the social complications of psychology online teaching.	Strongly disagree	5	6.7	0.04^*^
	Disagree	6	8.0	
	Neutral	21	28.0	
	Agree	25	33.3	
	Strongly agree	18	24.0	
Q12. One of the social life complications of psychology eLearning was lack of physical activities.	Strongly disagree	5	6.7	0.22
	Disagree	5	6.7	
	Neutral	24	32.0	
	Agree	14	18.7	
	Strongly agree	27	36.0	

The association between perception of online learning and its effect on students’ social health with sociodemographic data

In an analysis of factors influencing student perception of online teaching, gender and GPA did not show a significant difference in students’ perception (p-value > 0.05 for all; Table 4). Regarding the impact of online Blackboard platform teaching on students’ social health and psychology, gender showed a strong association (p-value < 0.05), as female students were significantly more likely to report social and psychological impacts than male students, with eight (21.6%) male students reporting affections of their social and psychological life compared to 29 (78.4%) female students (Chi-squared test; Table 5).

**Table 4:** The association between students’ online perception and sociodemographic data (n = 75) Pearson’s Chi-squared test.

Characteristic	Category	Poor perception No (44)	Good perception No (31)	p-value
Gender	Male	18 (41%)	9 (29%)	0.29
	Female	26 (59%)	22 (71%)	
GPA	Less than 2	2 (4.5%)	0 (0.0%)	0.585
	Between 2 and 2.5	5 (11.4%)	2 (6.5%)	
	Between 2.5 and 3	15 (34.1%)	9 (29%)	
	Between 3 and 3.5	12 (27.3%)	12 (38.7%)	
	Between 3.5 and 4	10 (22.7%)	8 (25.8%)	

**Table 5:** The association between online learning impact on students’ health and social life and sociodemographic data (n = 75) N (%), Pearson’s Chi-squared test, * p < 0.05 (significant).

Characteristic	Category	Poor social health No (37)	Good social health No (38)	p-value
Gender	Male	8 (21.6%)	19 (50%)	0.010^*^
	Female	29 (78.4%)	19 (50%)	
GPA	Less than 2	1 (2.7%)	1 (2.6%)	0.822
	Between 2 and 2.5	4 (10.8%)	3 (7.9%)	
	Between 2.5 and 3	11 (29.7%)	13 (34.2%)	
	Between 3 and 3.5	14 (37.8%)	10 (26.3%)	
	Between 3.5 and 4	7 (18.9%)	11 (28.9%)	

## Discussion

The aim of this study was to evaluate the perception of students to online teaching of a psychology course and its effect on the social life of medical students in Al-Qunfudhah Medical College, Umm Al-Qura University, Saudi Arabia. Due to the COVID-19 pandemic, universities worldwide transitioned from face-to-face teaching to online learning in 2020 [8,9]. One of the major universities in Saudi Arabia, Umm Al-Qura University, implemented Blackboard for online teaching to allow students to take live lectures and practical clinical sessions. The findings of this study documented that the students accepted the Blackboard teaching in a psychology course, but a large number of them reported that it affected their social life. Most of the students, 42 (56%), found themselves to be easily engaged in and interactive with online teaching. Previous studies reported the same findings [10,11]. Also, in parallel to the findings of our study, many studies documented the effectiveness of online teaching [12-14]. On the contrary, some studies reported that online teaching is as unsatisfactory as offline methods are for teaching [15,16]. When comparing the responses of students to online teaching versus face-to-face learning, we found a nearly equal response: 25 (33.3%) of them documented that online teaching was not as effective as face-to-face learning, whereas 27 (36%) agreed that online teaching was more effective than face-to-face learning. This difference can be regarded to the differences in student populations, types of the studded courses, or online platforms used.

In accordance with the results of our study, another study was performed at King Abdulaziz University, in Jedda, Saudia Arabia, and this study revealed that half of the medical students that participated in the study concluded that face-to-face teaching was similar to online teaching during COVID-19 outbreaks [14]. In another study performed at Imam Abdulrahman Bin Faisal University, 42% of medical students agreed that online teaching and face-to-face teaching were similar, whereas 22% of them remained neutral [17]. Other studies documented that 34% of students agreed that online teaching and face-to-face learning are equal in their effectiveness [18,19]. However, the study done by Dergham et al. [17] showed that the students preferred face-to-face teaching over online learning.

Regarding the impact of online teaching on students’ social life, our findings revealed that 47 (62.7%) students agreed that they had a headache during online teaching, 12% of them disagreed, and 25.3% were neutral. This result is parallel to the study done by Abou Hashish et al. [20], who reported that 65.72% of the participating medical students have headaches, whereas 34.28% of them do not suffer from headaches. Previous work suggested two possible causes of online-dependent headache. First, the effect of the brightness of the screen band on online devices. Second, the longtime exposure [21]. Additionally, Ranasinghe et al. [22] reported that longtime accommodation of the eyes may cause fatigue to extraocular muscles, leading to a headache [22]. Forty-eight (64%) and 43 (57.3%) suffered from anxiety and stress, respectively, during the psychology course online teaching through Blackboard, especially in female students, 29 (78.4%) (p < 0.05). This finding is in line with the study done by Pelucio et al. [23], who documented that medical students studying online an undergraduate psychology course suffered from anxiety 10.58 (4.98) and stress 7.66 (4.41). Also, in their study, Zhang and Ho [24] reported that one-third of 4,115 medical students from Wannan Medical College in China studying online during COVID-19 outbreaks suffered from stress and anxiety [24]. This can be regarded to social expectations or gender roles. Additionally, the results of our study reported that 43 (57.3%) suffered from social isolation and 41 (54.7%) were incapable of performing physical activities. This is in accordance with the study done by Kotera et al. [25], who reported that online students suffered from loneliness as well as the work done by Chu and Li [26], who documented that the compliance decreased by 24.31% during online teaching. Online teaching in our university more and more modified as IT unit in the university has made a marked changes in Blackboard platform overcoming the difficulties facing the students, e.g., interruption of internet flow, as it facilitates the recording of the lectures, so the student can see it at any time even with weak internet connection; moreover, the university established an online psychological clinic for supporting the students how suffering from social isolation or stress.

This work, however, had some limitations. First, a small sample size of the participants of one faculty of medicine; thus, the conclusion related to only a small population. Second, a self-reported measure of assessment resulted in bias in the quality of the data. Third, the questionnaire focused on just the perception of online teaching and its impact on the students’ social life, both of which were exposed to a bias of recall. Lastly, our cross-sectional study focused only on relation variables, not the cause-effect relationship. All these limitations can be resolved by more diverse samples or alternative data collection methods. So, future studies with larger sample sizes or longitudinal designs which better explore the cause-effect relationship between online learning and social impact are suggested.

## Conclusions

To sum up, the perception of medical students on online Blackboard teaching in a psychology course is positive because most of them see it as motivative, interactive, and easy to be engaged with the lectures and asking questions. Furthermore, most of the students documented that the medical staff was well-trained on the Blackboard platform. However, the students’ response is divided between online and face-to-face teaching as to which is better. On the controversy of the high perception of students toward online teaching, most of them reported that it affects their social life by interfering with their physical activities and socially isolating them, causing them to experience anxiety and stress disorders, especially among female students.
